# Impact of a Novel Insulin Management Service on Non-insulin Pharmaceutical Expenses

**DOI:** 10.36469/9783

**Published:** 2018-02-20

**Authors:** John E. Schneider, Anjani Parikh, Ivana Stojanovic

**Affiliations:** 1 Avalon Health Economics, Morristown, NJ, USA

**Keywords:** Economic Evaluation, Diabetes, Insulin Management Device

## Abstract

**Background:**

Studies have shown that improvements in glycemic control are associated with avoidance or delayed onset of diabetes complications, improvements in health-related quality of life, and reductions in diabetes-related health care costs. Clinical practice guidelines recommend maintaining a hemoglobin A1c (HbA1c) level less than 7%, but among type 2 diabetes patients using insulin, two-thirds have HbA1c above 7% and one-third have HbA1c above 9%.

**Objectives:**

This study examined the use of insulin management services to enable patients to optimize insulin dosing to achieve HbA1c targets and subsequently reduce health care costs. Cost savings may be achieved through reduced complications and hospitalizations, as well as reduced outpatient, physician, and clinic costs. This study quantified the reduction in pharmaceutical expenses related to the use of an enhanced insulin management service to improve glycemic control.

**Methods:**

Two hundred seventeen insulin-reliant patients were enrolled in the d-Nav® Insulin Guidance Service through a participating insurance group. A prospective cost analysis was conducted using data from enrolled patients who completed the first 90 days of follow up.

**Results:**

Of the 192 patients who completed the 90-day study period, 54 (28.13%) were prescribed one or more expensive medications at baseline, but 45 (83.33%) of those patients were eligible for medication discontinuation after 90 days. At baseline, the annual cost of expensive medications per patient was $7564 (CI: $5191–$9938) and $1483 (CI: −$1463–$4429) at 90 days (p<0.001). Direct savings from medication elimination was estimated to be $145 per patient per month (PPPM) or $1736 per patient per year (PPPY) for all patients and $514 PPPM/$6172 PPPY for the target group. Patients that completed the 90-day period significantly reduced HbA1c levels from 9.37% (CI:7.72%–11.03%) at baseline to 7.71% (CI: 6.70%–8.73%) (p<0.001). A total of 170 (88.54%) patients had improved HbA1c at 90 days.

**Conclusions:**

Use of the insulin guidance service achieved improved glycemic control by optimizing insulin dosing, which enabled most patients using the service to reduce or eliminate the use of expensive diabetes medications. Further study is needed to assess the impact of optimized insulin dosing on other diabetes-related health care costs in a usual practice setting.

## Introduction

Diabetes affects an estimated 30.3 million people in the U.S., representing nearly 10% of the general population; about 90–95% have type 2 diabetes (T2D).^1^ Numerous studies have shown that improvements in glycemic control are associated with avoidance or delayed onset of diabetes complications, improvements in patient health-related quality of life (HRQL), and reductions in diabetes-related health care costs.

Clinical practice guidelines for treating T2D recommend maintaining a hemoglobin A1c (HbA1c) level less than 7%, though benefits of lower HbA1c have also been noted.^2^ However, HbA1c targets often are difficult to maintain in usual practice. In the U.S., among type 2 diabetes patients using insulin, two-thirds have HbA1c above 7%, and one-third have HbA1c above 9%.^2^ Poor glycemic control in the T2D population is associated with a number of complications, including ischemic heart disease, congestive heart failure, stroke, foot ulcers, amputation, blindness, renal failure, and poor wound healing.^3^ The likelihood of T2D patients experiencing these complications is closely related to their degree of diabetes control, with the likelihood of complications increasing significantly for each 1% increase in HbA1c.^4^ For example, one study examining the relationship of glycemic control and hypoglycemic episodes over a 4-year period found that each 1% unit increase in average HbA1c from 9.3% was associated with a 31% increase in the risk of cardiovascular death and a 36% increase in the risk of stroke.^5^ In addition to reducing complications, improved glycemic control has also been shown to improve HRQL of diabetes patients, including improvement in symptom distress, cognitive functioning, general perceived health, and work productivity.^6^,^7^

The goal of insulin management services is to help patients optimize insulin dosing to enable them to achieve HbA1c targets and improve their HRQL. Improved glycemic control also reduces diabetes-related healthcare costs by reducing costs associated diabetes complications, such as hospitalizations and outpatient services. Optimized insulin dosing achieved through an insulin management service also may reduce overall drug costs by reducing the use of expensive diabetes medications. However, to date, the effect of insulin management services on the cost of care has only been examined within a decision model framework.^8^ The purpose of this study was to assess the ability of an insulin management service to reduce costs associated with the use of expensive diabetes drugs in a usual care setting within the U.S. health system.

## Background

### Glycemic Management

Good glycemic control is a well-accepted outcome for diabetes management, and clinical practice guidelines recommend a step-wise approach.^9^ Treatment typically begins with metformin, and if the HbA1c target is not achieved after 3 months, a second agent is added, such as sulfonylurea, thiazolidinedione, DPP-4 inhibitor, SGLT2 inhibitor, GLP-1 receptor agonist, or basal insulin. Due to increased risk of cardiovascular events, thiazolidinedione use has diminished in the past decade, and use of the other agents has increased.^10^,^11^ Specifically, costs associated with increased use of DPP-4 inhibitors, SGLT2 inhibitors, and GLP-1 receptor agonists has risen from $10 billion to $22 billion from 2002 to 2012.^12^ In addition, due to its progressive nature, many patients with T2D eventually require and benefit from insulin therapy, usually in combination with metformin. According to the standard of care, when a patient fails to reach their HbA1c target with dual therapy of metformin plus insulin, a third agent should be prescribed, such as thiazolidinedione, DPP-4 inhibitor, SGLT2 inhibitor, or GLP-1 receptor agonist.^9^ However, despite the wide availability of multiple classes of therapeutic agents, most insulin users have HbA1c well above the accepted targets and their diabetes is uncontrolled.^13^

### Diabetes and Insulin

Insulin is typically prescribed when a patient with advanced T2D becomes insulin deficient; it is one of the most commonly prescribed medications worldwide. In addition to insulin, many advanced T2D patients are prescribed a combination of oral anti-diabetics such as alpha-glucosidase inhibitors, biguanides, DPP-4 inhibitors, GLP-1 receptor agonists, meglitinides, SGLT-2, and sulfonylureas.^14^ Insulin formulations offer many benefits, including diverse pharmacodynamic profiles, no upper dosage limit, and only one source of toxicity (hypoglycemia). Despite the long-term availability and the potential advantages of insulin therapy, in practice its effectiveness is suboptimal. The average level of HbA1c for patients treated with insulin has not improved for decades, and frequent titration of insulin dosage is crucial to maintain optimal glycemic control and avoid hypoglycemia.^15^ Optimizing insulin dosing among insulin-reliant individuals with T2D has been shown to be a very effective means of lowering HbA1c and achieving clinical targets.^14^,^16^

### Optimizing Insulin Dosing

Multiple studies have emphasized the importance of frequent insulin titration in achieving glycemic goals.^14^,^16^–^18^ Dose titration, with frequent adjustments, has been shown to maximize treatment utility because metabolisms vary over time, beyond daily fluctuations in energy balance.^14^,^16^,^19^–^22^ However, the current structure of the health care system does not encourage (or in many cases permit) such frequent review of glucose data and corresponding timely insulin dosage modification.^19^ In usual practice, insulin dose adjustments are done every 90 to 180 days during an outpatient clinic visit.^16^ According to Khunti *et al*., “For most patients requiring insulin therapy, dose titration is carried out by physicians; however, evidence suggests that this process may not provide optimal glycemic management for patients.”^16^ To date, target HbA1c levels have only been consistently maintained in clinical trial settings with frequent insulin titration and monitoring.^21^ This improvement lasted only as long as periodic insulin adjustments were made. According to Harper *et al*., the magnitude of intra-individual variability in insulin requirements is considerable and explains both bouts of hyperglycemia and hypoglycemia in insulin users.^21^

### Insulin Management Service

The d-Nav^®^ Insulin Guidance Service was designed to optimize insulin therapy by providing safe and effective personalized insulin dosage recommendations to T2D patients without increasing the workload of referring physicians.^19^,^23^ This service is a combination of dedicated health care professionals and technology designed to simplify and enhance insulin therapy. Patients referred to the service receive training and support from a clinical team to help them use the accompanying d-Nav device, followed by ongoing supervision of the process, including clinical triaging to alert referring physicians in the case of abnormal disease progression. Patients use the device to monitor glucose levels before each injection and it then provides them with a personalized dose recommendation. By analyzing glucose patterns, the device automatically adjusts insulin dosage, as often as needed, to achieve and maintain optimal glycemic balance for each individual. This allows patients to adjust insulin therapy to fit their changing needs while preventing hypoglycemia. In several studies, the d-Nav Insulin Guidance Service has been shown to improve glycemic control, reduce hypoglycemia frequency, and reduce costs.^19^,^24^ As of May 2016, 727 patients have been referred to the service in Northern Ireland, and 533 patients have used it as of mid-May 2016. Among these active users, 47 patients have used the service for more than three years, and 103 have used it for more than two years. Average HbA1c improved from 9.5% (±1.6%) to just over 7%, and the lower rates were maintained throughout study periods. These results have been remarkably consistent in all studies of the service to date.^20^,^24^–^26^

## Methods

The objective of this study was to examine the cost differences within a cohort of T2D patients who use insulin, assessing the longitudinal changes from baseline [i.e., patient entering study treated under the “standard of care” (SOC)] to 90 days from starting the service. Patients were identified and referred by physicians from primary care and secondary care clinics in Southeastern Michigan. Adults (21–75 years of age) with T2D who were using insulin to manage their diabetes were eligible to participate if they had a HbA1c ≥ 7.5%, and were insured by Blue Cross Blue Shield of Michigan. Patients were excluded from the trial if they had a body mass index of ≥45 kg/m^2^; severe impairment of cardiac, hepatic, or renal functions; psychological or cognitive impairment; more than two episodes of severe hypoglycemia in the past year; or a history of hypoglycemia unawareness when glucose levels are ≤ 50 mg/dl. The d-Nav device used for this study is CE-marked and has been in commercial use in the United Kingdom since October 2012. The costs of non-insulin diabetes drugs were collected over the initial 90-day period, as were changes in HbA1c levels as a secondary endpoint.

### Study Design

For patients in the d-Nav arm, after they signed an informed consent form and were enrolled in the study, they received training on the proper use of the d-Nav device, and a baseline HbA1c level was obtained. At the end of the 90-day study period, patients returned to the care center for an HbA1c test. If the 90-day HbA1c was lower than the baseline HbA1c, then glycemic medications (other than insulin and metformin) were discontinued. All patients enrolled in the study were using one of four insulin regimens: (1) treated with the long-acting insulin analog Glargine (Lantus^®^); (2) treated with twice daily biphasic insulin (i.e., Humalog^®^ Mix 75/25, NovoLog^®^ Mix 70/30) or pre-mixed insulin (i.e., Humulin^®^ 70/30, Novolin^®^ 70/30); (3) treated with a short-acting insulin analog (i.e., Lispro-Humalog^®^, Aspart-NovoLog^®^, Glulisine-Apidra^®^) before each meal combined with the long-acting insulin analog Glargine; and (4) treated with a short-acting insulin analog before each meal utilizing an insulin/carbohydrate ratio for calculating their short-acting insulin doses, combined with the long-acting insulin analog Glargine.

After the initial visit was completed, patients were contacted via telephone at 2 days, 2 weeks, and 4 weeks to verify that patients were using d-Nav correctly and adhering to d-Nav recommended insulin dosing. Patients were withdrawn from the trial if (1) they wanted to stop utilizing the guidance service; (2) they were lost to follow-up; (3) switched insulin types to unsupported insulin; or (4) other health reasons. Patients who were withdrawn from the study were referred back to the SOC.

### Main Outcome Measures

The main outcome measure for this study was costs of care for a specified set of non-insulin diabetes related prescription drugs, with particular focus on the costs of GLP-1 receptor agonists drugs, DPP-4i drugs, and SGLT-2i drugs (hereinafter labeled as “expensive” branded diabetes medications). Diabetes drugs (other than insulin) used by each patient at baseline were obtained at baseline and at the end of the 90-day intervention period. Drug costs were calculated using the prevailing market price for each drug.^27^ The analysis focused on the mean value for pre- and post-levels of costs for expensive diabetes drugs, and the mean difference in these drug costs over the study period. The costs of insulin were excluded from this analysis for two reasons: (1) costs of insulin vary greatly by brand; and (2) insulin costs are volume dependent. Both parameters are outside of the control of the intervention. In addition, because the price of insulin is volume-based and there is no steady state for insulin dosage, the cost of insulin during the 9-month intervention may not reflect long- or short-term projected costs of insulin.^21^ In addition to costs, two secondary outcomes of interest were also assessed: change in HbA1c and patient satisfaction. To evaluate if patients achieved reduction in HbA1c levels during the study period, we determined the difference between baseline HbA1c and the HbA1c measured at 90 days for each patient using a two-tailed student’s t-test in Microsoft Excel. To assess patient satisfaction, surveys were mailed to everyone who completed the 90-day visit for the study. Participants were asked a series of questions regarding their perception and satisfaction with the service and were also asked how their satisfaction compared to the period of time prior to starting the service.

## Results

The study recruited a total of 217 patients, of which 192 patients completed the 90-day visit and were available for analysis. Reasons for withdrawal for the 25 patients that did not complete the 90-day visit included (1) withdrawn consent (n=12; 48%); (2) lost to follow-up (n=10; 40%); (3) switched insulin types to unsupported insulin (n=2; 8%); and (4) experienced a health complication unrelated to diabetes (n=1; 4%).

### Use of Expensive Medications

Of the 217 patients enrolled in the study, 57 (26.27%) were prescribed expensive medications at enrollment. Of the 57 patients, 3 (12.50%) withdrew before the 90-day visit. Therefore, of the 192 patients who completed the study, 54 (28.13%) were prescribed at least one expensive medication at time of enrollment. Of these, 27 (50.0%) were prescribed GLP-1 receptor agonists, 17 (31.5%) were prescribed DPP-4i drugs, and 13 (24.07%) were prescribed SGLT-2i drugs. Two were prescribed with more than one of these expensive medications, and one was prescribed with a combination of a DPP-4i and an SGLT-2i.

### Medication Discontinuation

Of the 192 patients that completed the 90-day visit, 170 (88.54%) had improved glycemic control and were therefore eligible for medication discontinuation. Of the 54 patients on one or more expensive medications, 45 (83.33%) were eligible for discontinuation based on improvement of their HbA1c. Among those 45 patients, the majority were taking GLP-1 or DPP-4 drugs (Table 1).

### Economic Effects

Based on discontinuation rates applied to data on drug costs, the direct savings from the elimination of medications are expected to be approximately $145 per participant per month (PPPM) or $1736 per participant per year (PPPY) for all patients and $514 PPPM/$6172 PPPY for the target group (n=54) (Table 2). There is a considerable range in costs depending on specific drug regimens. For example, for patients taking Victoza (n=12), monthly savings from discontinuation would be $832 PPPM and $9984 PPPY, and for patients prescribed Bydureon, the next most commonly prescribed brand-name drug (n=10), monthly savings from discontinuation would be $644 PPPM and $7728 PPPY (data not shown in table). Over the course of the study period, reductions in average 12-month medication cost savings for patients in the target group (n=54) was $7564 (CI: $5190–$9938) at setup and $1483 (CI: −$1463–$4429) at the end of the 90-day period (p<0.001). Reductions in average 12-month costs for all patients (n=192) at enrollment was $2362 (CI: −$1254–$5978) and $587 (CI: −$1178–$2352) (p<0.01) at 90 days. In sum, it is clear that the cost savings associated with improved glycemic control are substantial and likely to result in substantial savings over a relatively short period of time.

### Improved Glycemic Control

Average HbA1c for the 192 patients who completed the 90-day period improved from 9.37% (CI: 7.72–11.03) at baseline to 7.71% (CI: 6.70–8.73) by 1.66 percentage points (p<0.001). Average HbA1c for the target group patients on expensive medications (n=54) improved by 1.43 percentage points from 9.30% (CI: 7.7%–10.9%) at baseline to 7.86% (CI: 6.81%–8.91%) (p<0.001) at 90-days. A separate analysis was applied for the subgroup of patients who had HbA1c > 9% at baseline, usually referred to as “poorly controlled” diabetes. Of the 192 patients who completed the 90-day visit, 98 (51.04%) had a baseline HbA1c > 9%. Of these, 84 (85.71%) had a 90-day HbA1c ≤ 9%. This subgroup of 97 patients saw an average decrease of 2.86 percentage points in HbA1c, from 10.49% at baseline to 7.63% at the 90-day visit (p<0.001) (Table 3). Those patients who still had HbA1c>9% at the 90-day visit still experienced a smaller but significant decline of 0.85 percentage points.

### Patient Satisfaction

As a secondary outcome endpoint, patient satisfaction among users of the insulin management service was also assessed. Surveys were mailed to everyone who completed the 90-day visit for the study; 75 surveys were received in the first round of mailings, and 28 were received in the second round of mailings, for a total of 103 respondents. The average item-level response rate was greater than 98%. The questionnaire consisted of three sections: two asked participants about their overall satisfaction with their control of diabetes and the service, and a third asked about patient perception of diabetes management prior to starting the service. Participants were asked to score each question as follows: 1 = Strongly Agree; 2 = Somewhat Agree; 3 = Somewhat Disagree; 4 = Strongly Disagree; and DK = Don’t Know.

The mean score (and standard deviation) for key questions were as follows: “More satisfied with ability to manage diabetes” 1.31 (0.56); “Confident that d-Nav is using blood sugar results to change insulin dose” 1.26 (0.52); “Satisfied with decision to start using the d-Nav service” 1.17 (0.47); and “Important for patient to be able to continue using the d-Nav service” 1.22 (0.50). Overall, for all questions regarding the d-Nav study period, the vast majority of participants rated the service favorably, with the majority of responses either “Strongly Agree” or “Somewhat Agree.” For the questions regarding the period before using the service, participants generally ranked their disease management as less effective [e.g., “Satisfied with ability to manage diabetes” 3.08 (0.86)].

## Discussion

This study found that average annual cost of expensive medications by patients was $7564 (CI: $5191–$9938) and $1483 (CI: $−1463–$4429) (p<0.001). The direct savings from the elimination of medications alone estimated to be $145 PPPM or $1736 PPPY for all patients and $514 PPPM/$6172 PPPY in the target group of patients who were on expensive medications. Thus, for a health plan with 10,000 T2D “target group” patients, the estimated annual savings from medication discontinuation alone would be nearly $62 million. In addition to these cost savings, among the 192 patients who completed the 90-day period, there was a significant reduction in HbA1c from 9.37% ± 1.66% at baseline to 7.71% ± 1.02% (p<0.001) at the 90-day visit. This level of clinical improvement is likely to generate substantial additional savings beyond medication discontinuation.^4^,^7^,^28^,^29^ And it is clear from the results of the satisfaction survey that participants perceived that their disease management had improved markedly while using the service, with no reported disutility associated with use of the device and service.

These savings are likely an understatement of the savings that could be achieved by combining the insulin guidance service with a cost-efficient insulin utilization protocol. Note that 1000 units of Novolin 70/30 costs about $24, while 1000 units of Tresiba costs $308 and 1000 units of Humalog costs $353. Accordingly, if a patient takes 100 insulin units per day of basal-bolus insulin analogs, the monthly costs would be almost $1000. If that patient switches to Novolin 70/30 and doubles their insulin dose to 200 units per day, the monthly costs of insulin would still be only $144.^27^ Data provided in private communication by providers in the UK cohort of service users suggested that outcomes for patients on the service utilizing twice daily insulin therapy are slightly better than outcomes for service users who utilize basal-bolus insulin therapy, suggesting that implementing such an insulin utilization protocol would not compromise patient care.

Indirect effects are also likely to add to the direct service-related savings found in our study. Juarez *et al*., for example, suggested that approximately 30% of insulin users exhibit poorly controlled diabetes,^30^ and pay-for-performance measures such as Medicare STAR or HEDIS rewards providers if the vast majority of their patients have HbA1c ≤ 9%. Moreover, glycemic control is one of the standard measures that comprise the Center for Medicare & Medicaid Services’ (CMS) 5-star scoring system for performance-based bonuses to Medicare Advantage (MA) plans.^31^ Li and Doshi, for example, found that if a health plan is able to increase its composite CMS score by 1 star, that 1-star increase would be associated with an added 11 337 enrollees (p<0.001).^32^ This translates to a marginal enrollment increase of 12.3% (not reported in published paper; obtained via personal communication with authors). Thus, for a health plan with 1 000 000 enrollees, a 1-star increase is associated with a gain of 123 000 enrollees. Assuming an average annual insurance premium of $12 000, in this example the 1-star increase would lead to a $1.48 billion increase in revenue. This excludes the actual value of the bonus payment to the plan, which would be substantial: in 2016, for example, Cigna earned a bonus of more than $250 million.^33^ Another indirect effect is return-to-work and workplace productivity measures, such as presenteeism. While these outcomes were not directly assessed, they are likely to be positively impacted by the improved glycemic control.^34^–^36^

There are some expected limitations to the findings of this study. First, because this study involves the use of a medical device, patient adherence or lack thereof may impact HbA1c levels and overall study outcomes. Moreover, direct cost savings may also vary depending on cost of medication in different markets. Further follow-up research is recommended to better understand the long-term impact of the insulin guidance service on healthcare cost.

## Conclusions

An enhanced insulin management service provided substantial cost savings from improved glycemic control related to insulin dosing optimization and the associated reduction in utilization of expensive diabetes medications. We found that the direct savings from the elimination of expensive medications alone are expected to be approximately $145 PPPM or $1736 PPPY for all patients and $514 PPPM/$6172 PPPY for the target group. Marked improvements in glycemic control, as evidenced by the significant drop in HbA1c over the study period, are also likely to generate additional savings in diabetes-related medical care costs beyond savings from discontinuation of expensive drugs. These direct medical care savings are likely to understate total savings, such as savings associated with improved workplace productivity. In addition to cost saving, the use of an insulin utilization protocol to improved glycemic control could improve facility and health-plan level quality metrics, contributing to facility and health-plan revenues through higher payments based on quality performance.

## Figures and Tables

**Table 1:** Patients Prescribed and Taking Expensive Medications but Eligible for Discontinuation

Drug Class	% of Patients (n=54) (e)	% Eligible for Discontinuation (f)	Number of Prescriptions During Enrollment (g)	Number of Prescriptions Eligible for Discontinuation (h)
GLP-1 (a)	50.00%	85.19%	27	23
DPP-4 (b)(d)	31.48%	82.35%	17	14
SGLT-2 (c)(d)	24.07%	69.23%	13	9
**Total**	NA (i)	83.33%	57	46

Note: (a) GLP-1a drugs include Bydureon, Victoza, Byetta, and Trulicity; (b) DPP-4i drugs include Janumet and Januvia; (c) SGLT-2i drugs include Invokana, Farxiga, Invokamet, and Jardiance; (d) One patient was on Glyxambi, a combination DPP-4 and SGLT-2 drug and was counted for both drug classes for the purposes of this analysis; (e) Refers percentage of all patients who are on expensive drugs; (f) Out of all prescriptions categorized with this class of drugs, calculation of columns (h)/(g); (i) does not sum to 100% because of patients on more than one expensive drug.

**Table 2:** Comparison of Monthly and Annual Cost Savings for All Patients and Target Group Patients

Patient Group	Monthly Cost Savings (a)	Annual Cost Savings
All Patients (n=192)	$145	$1736
Target Group (n=54) (b)	$514	$6172

Notes: (a) Average monthly cost per patient; prices are reported by GoodRx.com and are based on market value as of October 2017; (b) Target Group represents cost savings for patients taking at least one expensive medication (either GLP-1a, DPP-4i, or SGLT-2i)

**Table 3:** Effect of d-Nav on Patient HbA1c Levels at 90-days (n=192)

Patient Cohort	Average HbA1c at Enrollment	Average HbA1c after 3 months of d-Nav	Percentage Point Difference (a)
All patients (n=192; 100.00%)	9.37%	7.71%	−1.66 (p<0.001)**
Target Group (n=54; 28.13%)	9.30%	7.86%	−1.44 (p<0.001)**
**HbA1c > 9% at baseline (n=98; 51.04%)**			
HbA1c ≤ 9% (n=84; 85.71%)	10.49%	7.63%	−2.86 (p<0.001)**
HbA1c > 9% (n=14; 14.29%)	11.05%	10.20%	−0.85 (p=0.04)*

Notes: Percentage point difference between baseline and 3-month assessment. See text.

* Significant at the 95% level

** Significant at the 99% level
